# DNA Damage in Inflammation-Related Carcinogenesis and Cancer Stem Cells

**DOI:** 10.1155/2013/387014

**Published:** 2013-12-05

**Authors:** Shiho Ohnishi, Ning Ma, Raynoo Thanan, Somchai Pinlaor, Olfat Hammam, Mariko Murata, Shosuke Kawanishi

**Affiliations:** ^1^Faculty of Pharmaceutical Sciences, Suzuka University of Medical Science, Suzuka 513-8670, Mie, Japan; ^2^Faculty of Health Science, Suzuka University of Medical Science, Suzuka 510-0293, Mie, Japan; ^3^Department of Biochemistry, Faculty of Medicine, Khon Kaen University, Khon Kaen 40002, Thailand; ^4^Department of Parasitology, Faculty of Medicine, Khon Kaen University, Khon Kaen 40002, Thailand; ^5^Liver Fluke and Cholangiocarcinoma Research Center, Faculty of Medicine, Khon Kaen University, Khon Kaen 40002, Thailand; ^6^Departments of Pathology and Urology, Theodor Bilharz Research Institute, Giza 12411, Egypt; ^7^Department of Environmental and Molecular Medicine, Mie University Graduate School of Medicine, Tsu 514-8507, Mie, Japan

## Abstract

Infection and chronic inflammation have been recognized as important factors for carcinogenesis. Under inflammatory conditions, reactive oxygen species (ROS) and reactive nitrogen species (RNS) are generated from inflammatory and epithelial cells and result in oxidative and nitrative DNA damage, such as 8-oxo-7,8-dihydro-2′-deoxyguanosine (8-oxodG) and 8-nitroguanine. The DNA damage can cause mutations and has been implicated in the initiation and/or promotion of inflammation-mediated carcinogenesis. It has been estimated that various infectious agents are carcinogenic to humans (IARC group 1), including parasites (*Schistosoma haematobium* (SH) and *Opisthorchis viverrini* (OV)), viruses (hepatitis C virus (HCV), human papillomavirus (HPV), and Epstein-Barr virus (EBV)), and bacterium *Helicobacter pylori* (HP). SH, OV, HCV, HPV, EBV, and HP are important risk factors for bladder cancer, cholangiocarcinoma, hepatocellular carcinoma, cervical cancer, nasopharyngeal carcinoma, and gastric cancer, respectively. We demonstrated that 8-nitroguanine was strongly formed via inducible nitric oxide synthase (iNOS) expression at these cancer sites of patients. Moreover, 8-nitroguanine was formed in Oct3/4-positive stem cells in SH-associated bladder cancer tissues and in Oct3/4- and CD133-positive stem cells in OV-associated cholangiocarcinoma tissues. Therefore, it is considered that oxidative and nitrative DNA damage in stem cells may play a key role in inflammation-related carcinogenesis.

## 1. DNA Damage in Inflammation-Related Carcinogenesis

Infection and chronic inflammation have been recognized as important risk factors for carcinogenesis and malignancies [1–3]. The International Agency for Research on Cancer (IARC) has estimated that approximately 18% of cancer cases worldwide are attributable to infectious diseases caused by bacteria, viruses, and parasites [4]. The burden of cancer caused by infectious agents is shown in Table 1. Inflammation can be induced not only by chronic infection, but also by many other physical, chemical, and immunological factors [5, 6]. It has been estimated that chronic inflammation accounts for approximately 25% of human cancers.

Under inflammatory conditions, reactive oxygen species (ROS) and reactive nitrogen species (RNS) are generated from inflammatory and epithelial cells [7]. ROS and RNS are capable of causing damage to various cellular constituents, such as nucleic acids, proteins, and lipids. ROS are generated from multiple sources, including inflammatory cells, carcinogenic chemicals and their metabolites, and the electron transport chain in mitochondria [2, 3]. ROS can induce the formation of oxidative DNA lesion products, including 8-oxo-7,8-dihydro-2′-deoxyguanosine (8-oxodG), which is considered to be mutagenic [7, 8]. During DNA synthesis, adenine is misincorporated opposite 8-oxodG, leading to G:C to T:A transversions [9].

Nitric oxide (NO) is synthesized by NO synthases. There are three isoforms: neuronal NO synthase (nNOS, also known as NOS1), inducible NO synthase (iNOS or NOS2), and endothelial NO synthase (eNOS or NOS3) [43, 44]. iNOS is activated to drastically generate NO in inflammatory and epithelial cells under inflammatory conditions, while eNOS and nNOS are constitutively expressed and produce relatively small amounts of NO. iNOS can be also upregulated by transcription factors such as NF-*κ*B, HIF1-*α*, STAT, and TNF-*α*. NF-*κ*B plays a central role in inflammation through its ability to induce transcription of proinflammatory genes, including iNOS, and functions as a tumor promoter in inflammation-associated cancer [45].

Excess NO production plays a crucial role in an enormous variety of pathological processes, including cancer [43]. NO reacts with superoxide (O_2_^•−^) to form peroxynitrite (ONOO^−^), a highly reactive species causing 8-oxodG and 8-nitroguanine [46, 47]. The reaction of guanine with ONOO^−^ forms 8-nitroguanine as the major compound [46], while adenine nitration is minor compared to its C8-oxidation [48]. Akaike et al. have demonstrated that 8-nitroguanine is formed via NO production associated with inflammation in mice with viral pneumonia [49]. 8-Nitroguanine is considered to be not only a marker of inflammation, but also a potential mutagenic DNA lesion, leading to carcinogenesis [50]. 8-Nitroguanine formed in DNA is chemically unstable and thus can be spontaneously released, resulting in the formation of an apurinic site [51]. The apurinic site can form a pair with adenine during DNA synthesis, leading to G:C to T:A transversions [52]. However, the discovery of translesion DNA polymerases and their role in the mutagenesis in living cells made this paradigm rather obsolete [53, 54]. AP sites are indeed mutagenic, but the A-rule does not really describe its mutagenic potential. Cells deficient in Rev1 and Rev3, subunits of DNA polymerase *ζ*, were hypersensitive to nitrative stress, and translesion DNA synthesis past apurinic sites mediated by this polymerase might contribute to extensive point mutations [55]. It has been reported that adenine is preferentially incorporated opposite 8-nitroguanine during DNA synthesis catalyzed by polymerase *η* and *κ*ΔC in a cell-free system, suggesting that G:C to T:A transversions can occur [56]. In the ONOO^−^-treated *supF *shuttle vector, which was replicated in host *Escherichia coli* cells, the majority of mutations occurred at G:C base pairs, predominantly involving G:C to T:A transversions [57]. Thus, 8-nitroguanine is a potentially mutagenic DNA lesion that can participate in initiation and promotion in infection-related carcinogenesis.

We have investigated the formation of 8-nitroguanine and 8-oxodG in various clinical specimens and animal models in relation to inflammation-related carcinogenesis, as summarized in Table 1. It is noteworthy that DNA damage was specifically induced at sites of carcinogenesis under chronic infection and various inflammatory conditions, as reviewed previously [2, 3]. It has been estimated that 11 infectious agents are carcinogenic to humans (Group 1) by IARC: parasites (*Schistosoma haematobium* (SH), *Opisthorchis viverrini* (OV), and *Clonorchis sinensis *(CS)), viruses (hepatitis B and C virus (HBV, and HCV), human papillomavirus (HPV), Epstein-Barr virus (EBV), human T-cell lymphotropic virus (HTLV-1), Kaposi's sarcoma herpesvirus (KSHV), and human immunodeficiency virus-1 (HIV-1)), and bacterium *Helicobacter pylori* (HP) [4, 58]. We demonstrated that 8-nitroguanine was strongly formed via iNOS expression at related cancer sites of SH, OV, HBV, HCV, HPV, EBV, and HP [2, 3, 10, 11]. The IARC classification of CS has been recently updated from 2A to 1, so we have not yet collected enough data for 8-nitroguanine. The mechanism of carcinogenesis by HTLV-1, KSHV, or HIV-1 seems not to be associative to inflammation. We could not observe 8-nitroguanine in leukaemia samples from patients infected with HTLV-1 (data not shown). 8-Nitroguanine was also formed in tissues from patients with inflammatory diseases, such as inflammatory bowel diseases (IBD), Lichen planus (LP), and Barrett's esophagus (BE) [3, 38]. Recently, we have reported that the formation of 8-nitroguanine and 8-oxodG increased significantly in the order of Barrett's esophageal adenocarcinoma > Barrett's esophagus > normal tissues. Treatment of BE patients with proton pump inhibitors (PPIs), which is expected to reduce the risk of Barrett's esophageal adenocarcinoma, suppressed these DNA lesions probably via activation of an antioxidant enzyme Mn-SOD [38]. Regarding inflammation-related carcinogenesis without infection, we describe the formation of 8-nitroguanine in lung tissues of mice intratracheally administered asbestos [42], although the precise mechanism of nitrative DNA damage remains to be clarified. Nitrative stress is involved in the asbestos-derived inflammatory response via myeloperoxidase [59–62] that plays a significant role in asbestos-induced carcinogenesis [63]. Interestingly, immunoreactivities of 8-nitroguanine, iNOS, and NF-*κ*B significantly increased in the order of carcinogenic potential: crocidolite (blue asbestos) > chrysotile (white asbestos) > control [42].

On the basis of our studies, various pathogenic factors induce inflammatory responses and the production of ROS and RNS * *from inflammatory and epithelial cells via iNOS expression, which is regulated by transcriptional factors including NF-*κ*B, STAT, and HIF-1*α* [2, 3]. Oxidative and nitrative stresses cause DNA damage, contributing to the accumulation of genetic alterations in tissues throughout the carcinogenic process. Particularly, 8-nitroguanine formation may participate in inflammation-related carcinogenesis as a common mechanism. Therefore, 8-nitroguanine could be used as a potential biomarker of inflammation-related carcinogenesis. Importantly, experimental evidence has suggested that 8-nitroguanine can lead to mutations, preferentially G:C to T:A transversions [46, 64], in addition to 8-oxodG [9, 65]. Indeed, G:C to T:A transversions have been observed in vivo in the ras gene [66] and the *p53 *tumor suppressor gene in lung and liver cancer [67, 68]. We also revealed that 8-nitroguanine and 8-oxodG were apparently formed in adenocarcinoma caused by mutated K-ras, by using conditional transgenic mice with K- ras^val12^ [69]. 8-Nitroguanine was colocalized with iNOS, NF-*κ*B, IKK, MAPK, MEK, and mutated K-ras, suggesting that oncogenic K-ras causes additional DNA damage via signaling pathways involving these molecules. It is noteworthy that K-ras mutation mediates not only cell overproliferation but also the accumulation of mutagenic DNA lesions, leading to carcinogenesis. These findings imply that DNA damage mediated by ROS and RNS may participate in carcinogenesis via activation of protooncogenes and inactivation of tumor suppressor genes.

## 2. Cancer Stem Cell Markers in Inflammation-Related Carcinogenesis

The cancer stem cell concept is widely accepted as important for overcoming cancer. Several studies have revealed that cancer cells show accumulation of mutations, genetic instability, and epigenetic change suggesting that cancer is also a disease of genes [70]. The most important question is how to generate cancer stem cells. Recently, many studies have reported on the expressions of stemness cell markers in various kinds of cancer. Table 1 summarizes possible markers of cancer stem cells, especially related to each inflammatory causative agent. We reported that 8-nitroguanine was strongly formed at all of these cancer sites from animals and patients with infectious agents, inflammatory diseases, and exposure to asbestos. Importantly, we also detected colocalization of 8-nitroguanine and stemness marker in infection-related carcinogenesis, as mentioned in the next section. On the basis of our recent studies, it is considered that chronic inflammation can increase mutagenic DNA lesions through ROS/RNS generation and can promote proliferation via stem cells activation for tissue regeneration. This idea is also supported by other papers about the association of cancer stem cells with infection and inflammation [71–74].

## 3. DNA Damage and Mutant Stem Cells Induced by *Schistosoma haematobium* Infection

Chronic infection with SH is associated with urinary bladder cancer [76]. Contact with contaminated freshwater is the major risk factor for infection. SH-associated bladder cancer is a common malignancy, especially in the Middle East and Africa. It is believed that the parasite's eggs in the host bladder result in irritation, eventual fibrosis, and chronic cystitis, leading to carcinogenesis. To investigate whether oxidative and nitrative DNA damage participate in inflammation-related carcinogenesis, we performed immunohistochemical analysis using bladder tissues obtained from cystitis and bladder cancer patients infected with SH. We demonstrated for the first time that 8-nitroguanine is formed in the tumors of bladder cancer patients with SH infection [10]. The formation of 8-nitroguanine and 8-oxodG was significantly higher in bladder cancer and cystitis tissues than in normal tissues. Oxidative DNA damage and SH infection were strongly correlated [10, 77]. iNOS expression was co-localized with NF-*κ*B in 8-nitroguanine-positive tumor cells from bladder cancer patients. NF-*κ*B can be activated by TNF-*α*, a major mediator of inflammation, which has been reported to increase in peripheral blood mononuclear cells stimulated by SH egg antigen [78]. It is reasonable to conclude that both 8-nitroguanine and 8-oxodG are formed by iNOS-mediated NO overproduction via NF-*κ*B activation, under SH-caused chronic inflammation.

**Figure 1:**
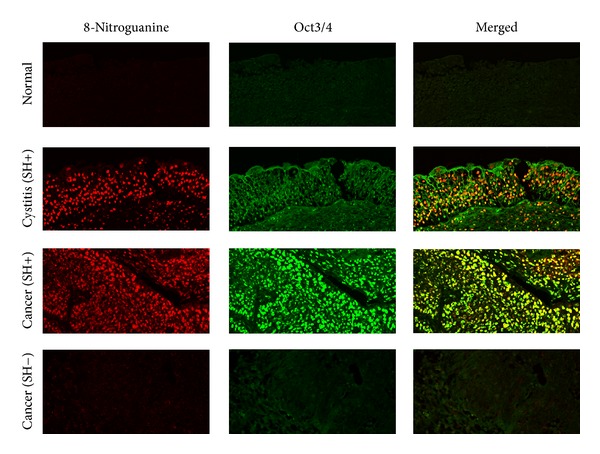
The formation of 8-nitroguanine (red) and the expression of Oct3/4 (green) were assessed by double immunofluorescence staining [10]. In the merged image, co-localization of 8-nitroguanine and Oct3/4 is indicated in yellow. Original magnification in all pictures is 200x (SH: *Schistosoma haematobium*). Formalin-fixed and paraffin-embedded biopsy and surgical specimens were obtained from normal subjects and patients with SH-induced cystitis and bladder cancer. Normal tissues and urinary bladder cancer tissues without SH infection were obtained from a commercial urinary bladder tissue array (Biomax.us, USA). Normal tissues with cystitis were excluded. SH-egg antigens in sera were detected by Sandwich ELISA assay [75]. This study was performed in accordance with the Ethical Guidelines for Epidemiological Research enacted by the Japanese government. Deparaffinized and antigen-retrieved sections were incubated first with 5% skim milk, and then with a rabbit polyclonal anti-8-nitroguanine antibody (2 *μ*g/mL, prepared as described previously [11]) and mouse monoclonal anti-Oct3/4 antibody (2 *μ*g/mL, Santa Cruz Biotechnology, CA, USA) overnight at room temperature. The sections were then incubated for 3 h with Alexa 594-labeled goat antibody against rabbit IgG and Alexa 488-labeled goat antibody against mouse IgG (each 1 : 400, Molecular Probes, Eugene, OR, USA).

A stemness marker Oct3/4 is generally expressed in pluripotent embryonic stem and germ cells [79]. Expression of Oct3/4 is reportedly necessary for maintaining the self-renewing, cancer stem-like, and chemoradioresistant properties of tumorigenic stem-like cell populations [80, 81] and is thus considered to play roles in the carcinogenesis process. Another stemness marker, CD44, has been identified as a cell surface marker associated with cancer stem cells in several types of tumors [82, 83], including urinary bladder cancer [84]. Expression of CD44v6, a splicing variant of CD44, is correlated with proliferation of poorly differentiated urothelial cells and the characteristic phenotype of tumor-initiating bladder cancer stem cells [85–87]. Our previous reports show that different risk factors induce different levels of expression of stemness markers in urinary bladder carcinoma. SH-induced urinary bladder cancer correlates with the expression of Oct3/4 [10], while urinary bladder cancer without the infection correlates with the expression of CD44v6 [11]. Moreover, 8-nitroguanine was formed in Oct3/4-positive stem cells in SH-associated cystitis and cancer tissues [10] as shown in Figure 1. Inflammation by SH infection may increase the number of mutant stem cells, in which iNOS-dependent DNA damage occurs via NF-*κ*B activation, leading to tumor development.

## 4. DNA Damage and Mutant Stem Cells Induced by *Opisthorchis viverrini *Infection

Chronic infection with the liver fluke OV is associated with cholangiocarcinomas [58]. Infection with this parasite is repeatedly caused by eating raw fish containing the infective stage of the fluke. We have demonstrated that 8-nitroguanine is formed in relation to inflammation-related carcinogenesis using an animal model [12–15]. 8-OxodG and 8-nitroguanine were formed in inflammatory cells and epithelium of bile ducts, and their formation increased in a manner dependent on infection times. The anthelmintic drug praziquantel dramatically diminished these DNA lesions and iNOS expression in OV-infected hamsters. Thus, repeated OV-infection can induce the iNOS expression in bile ducts and subsequently cause nitrative and oxidative damage to nucleic acids, which may participate in cholangiocarcinoma.

In our study with patients, the formation of 8-oxodG and 8-nitroguanine occurred to a much greater extent in cancerous tissue than in noncancerous tissue in intrahepatic cholangiocarcinoma patients, indicating that these DNA lesions contribute to tumor progression [16]. Our proteomic study showed that carbonylation of serotransferrin and heat shock protein 70 kDa protein 1 (HSP70.1) is significantly associated with poor prognoses [88]. Carbonylation of protein is an irreversible modification induced by oxidative stress. We have proposed that carbonylations of serotransferrin and HSP70.1 may induce oxidative stress by iron-accumulation and dysfunction of antioxidative property, leading to increased oxidative DNA damage and progression of cholangiocarcinoma.

Recently, we observed high expression and co-localization of hepatocyte marker and cholangiocyte marker in OV-associated cholangiocarcinoma patients, suggesting the involvement of stem cells in cholangiocarcinoma development [17]. Cholangiocarcinoma tissues with positive stemness markers (CD133 or Oct3/4) showed significantly lower expression of antioxidant enzyme Mn-SOD and significantly higher levels of 8-oxodG, 8-nitroguanine, and DNA damage response protein *γ*-H2AX. Moreover, CD133- and Oct3/4-positive cholangiocarcinoma patients had significant associations with tumor histology types, tumor stage, and poor prognoses. These findings suggest that CD133 and Oct3/4 in cholangiocarcinoma are highly associated with formation of DNA lesions, which may be involved in genetic instability and lead to tumor development with aggressive clinical features. In our study, proliferating cell nuclear antigen (PCNA) accumulated in the epithelium of bile ducts of hamsters after repeated OV infection, supporting the hypothesis that cell proliferation is promoted by inflammation-mediated DNA damage [14]. Inflammation by OV infection may increase the number of mutant stem cell, in which oxidative stresses, such as carbonylation of proteins and oxidative DNA damage, and cell proliferation are promoted, leading to progression of cholangiocarcinoma.

## 5. Conclusions

**Figure 2:**
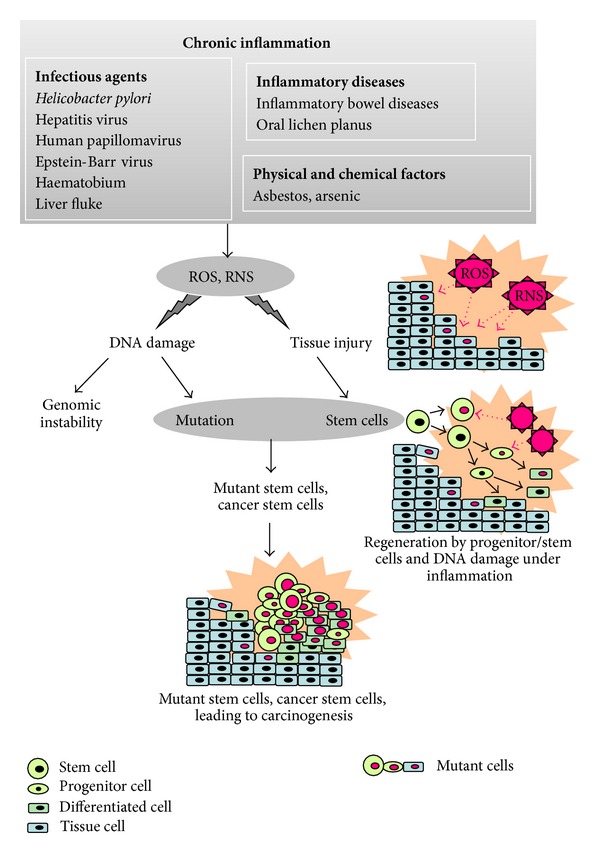
Postulated mechanism for generating cancer stem cells by inflammation.

Nitrative and oxidative DNA lesions with mutagenic properties are formed in various types of inflammation-related cancer tissues. We have proposed a mechanism for the generation of cancer stem cells by inflammation in Figure 2. Chronic inflammation by infectious agents, inflammatory diseases, and other factors causes various types of damage to nucleic acids, proteins, tissue, and so on, via ROS/RNS generation. Tissue injury under chronic inflammation may activate progenitor/stem cells for regeneration. In these cells, ROS/RNS from inflammation can cause multiple mutations, which may generate mutant stem cells and cancer stem cells, leading to carcinogenesis. Indeed, 8-nitroguanine was formed in stemness marker-positive cells in parasite-associated cancer tissues. The mechanism for generation of cancer stem cells will be explained by our ongoing studies on the formation of 8-nitroguanine in stem-like cells of target tissues associated with other inflammation-related cancers.

## Figures and Tables

**Table 1:** Possible markers for cancer stem cells in inflammation-related cancer.

Etiologic agent/pathologic condition		Associated cancer	Detection of 8-nitroguanine	Possible markers for cancer stem cells related to each cause [references]
Parasites	SH	Bladder cancer	Patients [10, 11]	Oct3/4 (patients with SH) [10]
				CD44v6 (patients without SH) [11]
	OV	Cholangiocarcinoma	Hamsters [12–15]	—
			Patients [16, 17]	CD133, Oct3/4 [17]

Viruses	HCV,	Hepatocellular carcinoma	Patients with HCV [18]	CK19 [19]
	HBV		Mice with HBV [DN]	Nanog, CD133 [20]
	HPV	Cervical carcinoma	Patients [21]	CK17 [22, 23]
				CD44 (HPV16) [24]
				Oct3/4 (HPV16) [25]
	EBV	Nasopharyngeal carcinoma	Patients [26]	LMP2A ([27] and a lot)
				LMP1, Bmi-1 [28]

Bacteria	HP	Gastric cancer	Patients [29, 30]	SALL4, KLF5 [31]
				KLF5 [32]
				LgR5 [33]

Inflammatory diseases	IBD	Colorectal cancer	Mice [34]	—
	LP	Oral squamous cell carcinoma	Patients [35]	Bmi-1 [36]
				KRT15 [37]
	BE	Barrett's esophageal adenocarcinoma	Patients [38]	Oct3/4 [39]
				CD133 [40]
				Musashi-1 [41]

Others	Asbestos	Mesothelioma, lung carcinoma	Mice [42]	—

SH: *Schistosoma haematobium*, OV: *Opisthorchis viverrini*, HCV: hepatitis C virus, HBV: hepatitis B virus, HPV: human papillomavirus, EBV: Epstein-Barr virus, HP: *Helicobacter pylori*, IBD: inflammatory bowel diseases, LP: lichen planus, BE: Barrett's esophagus, DN: data not shown.
